# Precious Data: Interim Report from the Jefferson Pancreas Tumor Registry

**DOI:** 10.1089/pancan.2018.0004

**Published:** 2018-06-01

**Authors:** Theresa P. Yeo, Harish Lavu, Avinoam Nevler, Jennifer Brumbaugh, Dominique Vicchairelli, Jordan M. Winter, Jonathan R. Brody, Charles J. Yeo

**Affiliations:** ^1^ Department of Surgery and the Jefferson Pancreas, Biliary and Related Cancer Center, Thomas Jefferson University, Sidney Kimmel Medical College, Philadelphia, Pennsylvania.; ^2^ Thomas Jefferson University, Jefferson College of Nursing, Philadelphia, Pennsylvania.; ^3^ The Dr. P. Borenstein Talpiot Medical Leadership Program, Chaim Sheba Medical Center, Israel.

**Keywords:** cancer registry, family history, pancreatic cancer, risk factors, quality of life

## Abstract

**Purpose:** The Jefferson Pancreas Tumor Registry (JPTR) is a voluntary hospital-based registry of persons with pancreas and related periampullary cancers, premalignant lesions, and nonaffected family members (NAFMs). The ultimate goals of the JPTR are to provide a link between family history, gene mutations, and precision medicine therapy, and to identify high-risk NAFMs for potential surveillance screening.

**Methods:** The JPTR is an Institutional Review Board approved longitudinal epidemiological study housed in the Department of Surgery at Thomas Jefferson University Hospital. Individuals who met the eligibility criteria and signed informed consent provide information on hereditary conditions, family history of cancers, environmental exposures, and occupational risk factors. Data are collected using a self-administered questionnaire, the electronic medical record, and the molecular analysis of tumor specimens.

**Results:** Established in 2008, >725 persons have enrolled in the JPTR. The cohort is mostly composed of sporadic pancreas cancer, with 13% of enrollees having familial pancreas cancer and a control group comprising nonaffected persons. Data from the registry have been utilized to inform clinical studies, molecular investigations, and to shed light on and gain insight into the lived experience of persons with these conditions.

**Conclusion:** The JPTR contains precious qualitative data and is an invaluable repository of information about persons with pancreatic and related tumors.

## Introduction

Population-based cancer registries were first established in Europe and North America in the 1940s and 1950s to generate statistics on the incidence of cancer in a defined population.^1^ The essential variables to be recorded by cancer registries include personal identification, gender, date of birth, address, ethnic group, incidence date, site of cancer, histology, tumor behavior (benign or malignant), and the source of information.^1^ Recommended or desirable additional variables are date of last contact, survival status, stage at diagnosis, and treatment. There are numerous types of cancer registries including those that are nationally, state, and regionally mandated; cancer-type specific; those focused on end results; and voluntary hospital-based registries. Since years lived after a cancer diagnosis is a crude measure of outcome, refined measures have evolved that include disease-free survival and evaluating the quality of life of participants.^1^The Jefferson Pancreas Tumor Registry (JPTR), a voluntary, hospital-based registry housed in the Department of Surgery (DoS), is a longitudinal epidemiological study of individuals with pancreatic cancer (PC) and related periampullary malignancies, including ampullary cancer, bile duct cancer, duodenal cancer, and entities such as intraductal papillary mucinous neoplasms (IPMNs), and pancreatic neuroendocrine tumors (PNETs).

The JPTR was established in 2008 at the Thomas Jefferson University Hospital (TJUH) in Philadelphia, Pennsylvania (PA). It was modeled after The National Familial Pancreas Tumor Registry at The Johns Hopkins Hospital, in Baltimore, Maryland, with input from an advisory panel. The Thomas Jefferson University Institutional Review Board (IRB) has approved the JPTR and it undergoes yearly Continuing IRB Review. Data from the registry have been utilized to inform clinical studies, treatment strategies, molecular investigations, and to cast light on the lived experience of persons with these conditions. Provided herein is a short descriptive review of the cohort, the scholarly activities of the JPTR to date, and the results from our 2016–2017 follow-up using the Annual Update and the Survivor Survey.

## Methods

The aims of the JPTR are threefold: (1) to develop a data base of self-reported information on hereditary conditions, family history of cancers, environmental exposures, and occupational risk factors; (2) to provide a link between gene mutations, family history, and precision medicine therapy (in conjunction with the ongoing Jefferson Tissue Banking Study); and (3) to identify high-risk nonaffected family members (NAFMs) for potential surveillance screening. Information on sociodemographic background, lifestyle factors, family history of any cancers, known inherited genetic syndromes, longest-held occupation, and 20 known environmental risk factors, including tobacco exposure and industrial carcinogens, is collected through a self-administered questionnaire and entered into a password-protected access database maintained by the JPTR Co-Director and the Coordinator. The questionnaire may be accessed either directly from a Co-Investigator at our institution or through the DoS website. Data for analysis are collected through hard copies of JPTR questionnaires, electronic medical record, operative notes, molecular analysis, and pathology reports. Family genograms are created using Progeny 8^®^ software.^2^

All persons with known or presumed PC and related cancers and premalignant lesions, who are 18 years of age or older, are eligible to participate. NAFMs of the index case are encouraged to enroll as well and serve as a control group. Both groups must provide written informed consent before completing the questionnaire. Recruitment for the registry may occur at an initial outpatient consultation, after surgery, through the DoS website, or at specialized patient symposiums at our institution. Registrants are provided with printed information on the registry and given the questionnaire, which can be returned through mail in a self-addressed stamped envelope. Several Jefferson-affiliated IRB hospital sites in PA and New Jersey also enroll a limited number of patients with PC at their facilities. These patients have typically received either chemotherapy or radiation therapy (RT) at that institution but have not had surgery there. JPTR registrants are classified as either (a) familial pancreatic cancer (FPC) (defined as one or more first degree relative with PC in addition to the index case),^3^ (b) sporadic pancreatic cancer (SPC) (index case only has PC), which is consistent with other established cancer registries, or (c) control group, (defined as NAFMs). It should be noted that the control group is dynamic in that members may later be diagnosed with either FPC or SPC and reclassified as necessary.

Annual follow-up of JPTR registrants is conducted by three methods: (1) an Annual Update is mailed directly to all registrants or to a designated relative or next-of-kin. The update inquires about current health conditions, the recurrence of PC, or the development of other cancers or medical conditions in the index case, registrant, and in other family members, and (2) a unique specialized Survivor Survey is mailed to those registrants presumed to be living. This survey includes probing questions on quality-of-life issues, ongoing disease, or postsurgery complications, and individual reflections on treatment decision-making, and (3) the survivorship status of the cohort is documented through the use of Internet searches for published obituaries, reports on the annual surveys, and by families notifying us directly by letter, e-mail, or phone call of a death.

Our newsletter, *The Jefferson Pancreas Tumor Registry Update,* is published each year and is mailed to all registrants and to all listed contact persons.^4^ The newsletter is a benefit to those enrolled and features an uplifting survivor story and provides an update on the current status and results of ongoing PC research studies at Jefferson. In addition, the newsletter serves as a venue to report on the annual Sidney Kimmel Cancer Center *Jefferson Pancreas Cancer and Related Diseases Symposium* and to display the annual PC survivor group photo. The symposium, held every November (Pancreas Cancer Awareness Month), was initiated by the DoS in 2005 as a community outreach effort for PC and related cancer survivors, their families, and interested community members. Over the past 12 years, it has grown in magnitude, focus, and influence. It is a highly anticipated event with well >200 attendees. The complimentary program features a gastrointestinal-friendly breakfast followed by short reports on the current state-of-the-science in pancreas cancer research at Jefferson and interesting clinical topics. The symposium is unique and likely the largest of its kind in the United States. These activities contribute to an enhanced feeling of community for PC survivors and their loved ones. Attendees receive a mailing from the Thomas Jefferson University Office of Institutional Advancement and many make a contribution to support pancreas cancer research.

## Results

### Demographics

Since 2008, ∼23% of eligible patients at TJUH have been recruited to participate in the JPTR. Seven hundred eighty-four individuals have enrolled in the JPTR, of which 707 have mostly complete and useable information. The majority of registrants (67%) have SPC, 13% are FPC cases, NAFMs (controls) constitute 11%, and the remaining 8% have other biliary conditions or nonpancreas cancers. (Selected clinical characteristics of the cohort are shown in Table 1).

**Table 1.** **2008–2018 Jefferson Pancreas Tumor Registry Registrant Characteristics (*N* = 707), *n* (%)**

Sporadic PC	485 (67%)
Familial PC	92 (13%)
Controls (NAFMs)	90 (13%)
Related conditions	40 (5%)
High-risk NAFMs	82 (11%)
Gender: women	51%
Median age: (range 21–90 years)	66
Race: White	92%
African American	3%
Asian, Hispanic	2%
Unknown	3%
Ashkenazi heritage: mother	57 (8%)
Father	59 (8%)
Both parents	48 (7%)
Smoking (current or former)	315/688 (46%)
Diabetes	165/586 (28%)
Top exposures: asbestos	74/682 (11%)
Pesticides/herbicides	72/682 (10%)
Residential radon	50/682 (7%)
Heavy wood dust	43/682 (6%)

Related conditions: duodenal cancer, bile duct cancer, IPMNs, pancreatitis, cysts. Denominators vary based on number that answered the question.

PC, pancreatic cancer; IPMN, intrapapillary mucinous neoplasm; NAFM, nonaffected family member.

Ninety-two FPC cases were identified by defining FPC as the index case plus one first-degree family member with PC. Using a more stringent definition of family history as the index case plus two first-degree family members and at least one second-degree relative with PC, 26 (28%) cases were identified. Thirty-six (39%) of the FPC cases also reported between three and seven other types of cancers in their first- or second-degree relatives. There was one FPC case who had a family history of hereditary pancreatitis, otherwise no high-risk familial syndromes were identified. Genetic testing for specific gene mutations was not conducted. The characteristics of FPC cases and contributory risk factors for PC are shown in Table 2. Cigarette smoking was reported by 45% of the FPC cases, attesting to the synergistic effect of smoking and a familial history of PC. As reported by Schenk, a family history of PC or ever-smoking cigarettes doubles one's risk of developing PC (relative risk 2.0), and for current smokers who were also related to someone with PC, the relative risk of PC jumped to 8.23.^5^

**Table 2.** **Characteristics of Familial Pancreatic Cases (*N* = 92)**

	N (%)
Index case and one first-degree relative with PC	92
Index case and two first-degree and at least one second-degree relatives with PC	26 (28)
Other contributory risk factors	
Smoking	41 (45)
Second hand smoke exposure	12 (13)
Diabetes	9 (10)
Ashkenazi heritage	7 (8)

The demographic background of the JPTR is consistent with other published PC registry data,^6,7^ although they differ somewhat from nationally published statistics^8^ in that the median age of those enrolled is 66 years, the sample is 92% white, 3% African American, 2% Asian or Hispanic, and 3% of unknown race or ethnicity. Men (49%) and women (51%) have near equal representation. Almost half of the cohort (46%) were either current or former cigarette smokers. Twenty-eight percent of the registrants had diabetes at the time of enrollment. Studies have shown that new onset of diabetes in persons 50 years of age or older, coupled with weight loss and long-term smoking, should raise the index of suspicion by primary care clinicians about an underlying cancer and warrant screening by magnetic resonance imaging/magnetic resonance cholangiopancreatography (MRI/MRCP).^9,10^ Among PC patients undergoing either pancreaticoduodenectomy or distal pancreatectomy, the prevalence of preoperative diabetes ranged from 25% to 29%.^10^

Asbestos is the most commonly reported occupational or environmental exposure reported followed by pesticides and herbicides, residential radon, and wood dust. This is consistent with previously reported findings on occupational and environmental risks for PC.^3^ However, it should be noted that most individuals are not aware of potentially carcinogenic exposures throughout their lifetime and answer “Don't Know” to the list of 20 known exposures associated with PC.

Twelve percent of the registrants were either Jewish or reported Ashkenazi Jewish heritage in one parent (8%) or both parents (7%). Ashkenazi heritage has been reported to increase the risk of PC 10-fold when there is a germline *BRCA2*, 6174del1T mutation.^11^ In addition, in unselected Jewish populations, the prevalence of the three founder mutations in *BRCA1*(185delAG, 5382insC) or *BRCA2* (6174delT) was 5.5%, higher than previously known.^12^ Identifying *BRCA1/2* mutations has important therapeutic implications as poly (ADP-ribose) polymerase inhibitors may have significant activity in *BRCA*-associated PC.^13^ Although ascertainment bias is present in the JPTR, we are nonetheless reaching an Ashkenazi and Jewish patient population that may benefit from participation in the JPTR in terms of referral for surveillance screening and family consultation with a genetic counselor.

Eight years of JPTR cohort follow-up has yielded at least one SPC case in which a second family member developed PC during the follow-up period. This is consistent with the low incidence of FPC (5–10%)^3^ in the general population.

### Molecular risk factors

The linkage of cancer registry data to a pre- or coexisting database is useful in the study of molecular risk factors. In conjunction with our ongoing Jefferson Tissue Banking study, JPTR data have attempted to associate single nucleotide polymorphisms (SNPs) with pathological features or family history of cancers and/or germline mutations. (This requires that patients undergoing surgery at TJUH have given consent for both studies.) A recent article by our laboratory described a correlation between a functional SNP in an immune regulator gene encoding the enzyme indoleamine-2,3-dioxygenase-2 (known as *IDO2*) and the risk of FPC.^14^ PCR amplification of DNA extracted from resected specimens stored and annotated in our biobank was used to identify a wild-type, heterozygous, and a homozygous *IDO2* genotype, which has been previously shown to correlate with decreased enzymatic functionality.^14,15^ In this preliminary, single-institution study, the homozygous genotype (*IDO2*) was associated with an increased risk of FPC as compared with a control group.^14^

We also identified SNPs (insertions and deletions [INDELs]) embedded within the 3′UTR of the mitotic kinase checkpoint inhibitor gene, WEE1.^*^ This site is a previously published HuR binding site.^15^ Tumor samples from resected patients with pancreatic ductal adenocarcinoma in the FPC group from the JPTR were analyzed using standard gene amplification techniques to identify wild-type, heterozygous, and homozygous WEE1 allelic alterations. Index PC cases with the 12T allele were significantly more likely to have first-degree relatives with Lynch-type cancers (colorectal, endometrial, gastric, and ovarian) as compared with cases without a family history of these cancers.^*^ Both the IDO2 and the WEE1 SNPs may have significant implications for screening high-risk individuals and may also have value as a predictive marker for therapy using novel immune checkpoint inhibitors or DNA damaging therapies.

### Decision counseling

Recruitment to cancer registries can be challenging in the era of Internet access due to fears of confidentiality. Thus in 2014, the JPTR served as the setting for a study designed to assess the impact of the Decision Counseling Program^®^ (DCP), a shared decision-making software program, on recruitment to the JPTR. The study was the first to evaluate the impact of a DCP specifically on the registry enrollment of patients with pancreas cancer. Meyers et al. found that 80% of PC patients who received in-person decision counseling enrolled in the JPTR as compared with only 47% of PC patients who had telephone counseling.^16^ Altruism was the most frequently cited motivating factor for participating by both those who enrolled and those who did not enroll. This study added to the literature on cancer registries by identifying obstacles that led to the decision to decline enrollment in the JPTR: that is, the complexity of the registration process, feeling overwhelmed by the process of recovering from surgery, and worry about the confidentiality of their information. Based on these results, we have implemented a more personalized enrollment procedure and highlight data security and the confidential nature of the JPTR information.

### Identification of high-risk NAFMs

A cohort of high-risk NAFMs (*n* = 78) have been identified using the following criteria: first and second-degree relatives with confirmed PC, known high-risk familial syndromes (hereditary pancreatitis, hereditary nonpolyposis colorectal cancer [Lynch syndrome], hereditary breast and ovarian cancer, familial atypical multiple mole melanoma [FAMMM], Peutz–Jeghers [PJ] syndrome), known BRCA1 or BRCA2 mutations, family or personal history of ovarian cancer, cigarette smoking, pack-years smoked, second-hand smoke exposure, and exposure to asbestos, pesticides, herbicides, or residential radon, wood dust, and other known carcinogens. Canto et al. have shown some benefit in screening high-risk individuals through endoscopic ultrasound (EUS) and computed tomography screening.^17,18^ The diagnostic yield for finding actionable pancreatic neoplasms was only 5.3% (2/38) in a 2004 study of high-risk individuals with two or three family members with PC and one patient with PJ syndrome.^17^ A 2006 prospective study of 87 high-risk individuals and 149 controls had a 10% diagnostic yield using EUS at baseline and at 12 months.^18^ Thus, we encourage high-risk NAFMs to seek consultation with a genetic counselor and a gastroenterology pancreas specialist (skilled in EUS) to undergo assessment and a screening workup consistent with the 2012 International Cancer of the Pancreas Screening (CAPS) Consortium recommendations.^19^

### Annual tracking

We have been conducting annual follow-up on the JPTR cohort since 2010. In 2015, the response rate was a remarkable 77%.^7^ The most recent data from 2016 to 17 are presented in Table 3. The Annual Update was sent to 364 persons: 199 responses were received (55%), with 165 persons (45%) either not responding or the letter was returned to sender. No new cases of PC were reported. Concurrently, the annual Survivor Survey was mailed to 200 registrants, presumed to be living. We received 101 responses for a 51% return rate. Of those persons responding, 71% had a cancer, (81% of which was PC, 8% periampullary, 7% bile duct, and 4% duodenal). Twenty-nine percent had a benign condition including IPMNs, pancreas cysts, pancreatitis, and PNETs. The majority of respondents had undergone surgery (96%) for either a malignant or benign neoplasm. A subset received chemotherapy and/or RT after surgery, whereas four persons did not undergo surgery, but did undergo chemotherapy. Ninety-six percent of the respondents were alive, with five spouses completing the survey as a proxy for their deceased spouse. More women (52%) than men returned the Survivor Survey.

**Table 3.** **2016–2017 Jefferson Pancreas Tumor Registry Annual Update and Survivor Survey Results**

Surveys mailed	N (%)
(1) Annual Update	364
Completed	199 (55)
No response or RTS	165
(2) Survivor survey	200
Completed	101 (51)
No response or RTS	99
Survivor survey results (*n* = 101)	
Gender: women	52 (52)
Confirmed status: alive	96 (95)
Condition: cancer (*n*):	72 (71)
Pancreas	58
Periampullary	6
Bile duct	5
Duodenal	3
Not cancer (*n*):	29 (29)
Pancreas cyst	19
Pancreatitis	11
IPMN	8
Pancreas endocrine tumor	2
Treatment: surgery only	97 (96)
Surgery + CT	57 (58)
Surgery + CT + RT	33 (34)
CT only	4 (4)

RTS, returned to sender; IPMN, intrapapillary mucinous neoplasm; CT, chemotherapy; RT, radiation therapy.

### Quality of life

As part of the Survivor Survey, patients were queried about their perceived quality of life and persistent symptoms (Table 4). On a scale of 1–5, where 1 represents poor quality and 5 represents excellent, quality of life was rated 4.2/5. The ability to perform activities of daily living on a scale of 1–4 was rated 3.8/4. Seventy-six percent of respondents were walking regularly and 46% reported that they exercised regularly. The most common activities performed were in rank order: walking, lifting weights, “going to the gym,” golfing, elliptical use, biking, use of a treadmill, and going to physical therapy. Eighty patients reported being involved in 22 additional types of physical activity.

**Table 4.** **2016–2017 Jefferson Pancreas Tumor Registry Quality-of-Life Findings**

Rate your quality of life		4.2/5 (1–5, 1 = poor, 5 = excellent)		
Ability to do activities of daily living		3.8/4 (1–4, 1 = poor, 4 = excellent)		
Top three persistent symptoms (*n* = 101) (%):		Fatigue (38)		
Diarrhea (26)				
Bloating (20)				
Walk regularly (*n*%):		Yes = 63 (76)	No = 20	
Exercise regularly (*n*%):		Yes = 38 (46)	No = 42	
Medical marijuana for symptoms (*n*%):		Yes = 7 (7)	No = 87	
Require pancreatic enzymes (*n*%):		Yes = 47 (51)	No = 46	
Return to work after treatment (*n*%):		Yes = 43 (43)	No = 8	N/A = 50
Most commonly reported physical activities in rank order (*n* = 93):				
1. Walking		5. Elliptical		
2. Lifting weights		6. Biking		
3. Going to the gym		7. Treadmill		
4. Golfing		8. Physical therapy		
22 Other activities reported by respondents (*n* = 80):				
Balance work	Core work	Occupational therapy	Water aerobics	
Bow flex	Fishing	Pilates	Work on ranch	
Cancer rehabilitation	Gardening	Skiing	Yoga	
Cardiac rehabilitation	Hiking	Spinning	Zumba	
“Cardio”	Housework	Swimming		
Chopping wood	Hunting	Tennis		

N/A, not applicable, retired at time of diagnosis. Denominators vary based on number that answered the question.

Persistent bothersome symptoms were reported by respondents as follows: fatigue (38%), diarrhea (26%), bloating (20%), weight loss (17%), and pain and poor appetite (14% each). These particular symptoms have frequently been reported in the PC literature, with fatigue being the most persistent and bothersome adverse effect.^20^ Half of the respondents still required exogenous pancreatic enzymes on a daily basis. Only 7% reported using medical marijuana to deal with pain issues.

This year, we added a question on one's ability to return to work. Fifty respondents were retired at the time of diagnosis, 43 were able to return to their preillness occupation, 6 people reported inability to perform their previous job, and 2 people reported that they were unable to get a position in their field because of perceived discrimination by employers regarding their cancer diagnosis. This perception merits further investigation in future surveys.

### Satisfaction with decision-making choices

Survivor Survey respondents (*n* = 101) were also asked a series of questions designed to determine their satisfaction with the decisions that they had made regarding their treatment options (Table 5). To clarify, 72 of the respondents had either pancreatic, ampullary, bile duct, or duodenal cancers and 29 had nonmalignant conditions at the time they entered the JPTR. Ninety-seven of these respondents underwent surgery, the PPPD being the most common operation. The majority of these patients also opted for adjuvant chemotherapy and/or RT. Four patients in the sample had cancer but did not undergo surgery; they received only chemotherapy. The stage of disease at the time of the Survivor Survey was not known.

**Table 5.** **Satisfaction with Decision-Making (*N* = 101)**

If you had to make the decision over, would you have surgery again?	Yes = 94%	No = 2%	
Would you have chemotherapy again?	Yes = 60%	No = 9%	
Would you have radiation therapy again?	Yes = 36%	No = 21	
If your cancer came back would you have treatment again?	Yes = 76%	No = 2%	Maybe = 2%
Have you received palliative care?	Yes = 5%	No = 76%	

Denominator = 101; not all respondents answered every question, thus does not sum to 100%.

Surgery was clearly felt to be the most valuable treatment choice with 94% responding “yes” they would have surgery again, 60% responding that they would have chemotherapy again, and only 36% felt that they would have RT again. Cancer survivors were also asked a hypothetical question about treatment if their cancer returned. Seventy-six respondents replied that they would have surgery again, two said “no,” and two replied “maybe,” indicating that they would have to consider “other factors.” More than 75 respondents were aware of palliative care therapies and 5 persons reported receiving palliative care at the time of the survey.

### Strengths of the study

This report provides a detailed report on our institutional experience and a paradigm for establishing a specialized registry. The annual longitudinal follow-up on a cohort of PC and other survivors offers us the opportunity to chart the natural history of the disease. The ability to correlate the tumor biology with family history is a window into actionable molecular targets and recommendations for family member genetic testing.

### Limitations of the study

Registry data are subject to inherent limitations that restrict its generalizability. Quality control problems exist, such as incomplete and inaccurate data. Most of the demographic information is self-reported and unverified, and measures of occupational and environmental exposures are not quantifiable. Owing to the various methods by which one can join the JPTR, we do not collect information on the stage of disease at the time of enrollment.

Issues that are more difficult are potential nonconcordance between pathologists about the diagnosis of pancreas cancer, and the fact that persons can have more than one cancer and other comorbidities that influence survival and quality of life. Enrollment in a private disease-specific registry such as the JPTR is voluntary and, therefore, does not represent the overall U.S. epidemiology of PC,^5^ particularly with regard to race and ethnic demographics. In the case of the JPTR, this is a geographic limitation and solely a function of patients who chose to come to our center and not any intentional selection bias. All patients meeting the eligibility criteria are encouraged to participate.

In addition, as is found across multiple institutions, biobanking and the integrity of the tissue preserved could be a limitation for specific molecular profiling studies performed in a retrospective manner, particularly with analytes susceptible to degradation, such as RNA.

## Discussion

We report an above-average response rate (exceptional at 77% in 2015) to our two annual JPTR follow-up surveys, which indicates a committed patient population and speaks to the quality and maintenance of the registry database. JPTR enrollees report a high degree of satisfaction with their decision-making choices despite some having conditions with a poor prognosis. This indicates the importance to patients of having input and a degree of control about their treatment options. More patients this year were aware of palliative care and a small number were receiving it. A high quality of life was reported by both patients with cancer and those treated for premalignant and benign disease. Survivors remained physically active despite lingering adverse effects—fatigue and gastrointestinal problems being the most prevalent. Overall this subset of JPTR registrants is a high-functioning group.

The availability and use of registry data such as the JPTR enhance the ability to investigate potentially important heritable gene mutations and SNPs that may be actionable. Evaluating this type of data is important when advising patients on treatment options and when providing advice to individuals regarding screening benefits and costs.

In addition, the JPTR offers a rare opportunity to glimpse into the lived experience of those who have pancreas, biliary, and other related cancers and conditions in a prospective longitudinal manner. As such, this is precious data. Cancer registries have not routinely tracked quality of life (such as long-term side effects of treatment and work disability) between diagnosis and death because it requires complex data collection by direct patient contact.^1^ The JPTR is unique in that through our Annual Update and Survivor Survey, we have documented the lived experience of persons with PC. Our future plans for the registry are to continue to evolve and query survivors about matters that are pertinent and relevant to their lives.

Scientifically, serial follow-up and linking individuals with pancreas and related cancers to genetic backgrounds (familial and ancestry) along with environmental landscapes will continue to allow us to gain insights into this disease. For example, the JPTR may allow us to address the following: How can we better identify high-risk individuals? What life style changes may help curtail the development of this disease? Which patients are best served by surgery and which patients should receive other therapeutic interventions?

## Conclusions

This interim report of the JPTR describes the utility of a specialized cancer registry for scientific discovery, monitoring disease status, determining survivorship, and identifying high-risk NAFMs for surveillance and screening. The JPTR is an invaluable resource at our institution for its ability to provide a link for families and their nonaffected relatives to monitor research progress related to their conditions. As researchers, it enables us to contact patients to determine comorbidities and survival status. As a community resource, it provides continuity between patients and their family members and the surgeons, nurses, oncologists, and scientists who are making unique discoveries about the biology of the diseases and innovative therapies.

## Abbreviations Used

DoS: Department of Surgery
EUS: endoscopic ultrasound
FPC: familial pancreatic cancer
IPMNs: intraductal papillary mucinous neoplasms
IRB: Institutional Review Board
JPTR: Jefferson Pancreas Tumor Registry
NAFMs: nonaffected family members
PC: pancreatic cancer
PNETs: pancreatic neuroendocrine tumors
RT: radiation therapy
SNPs: single nucleotide polymorphisms
SPC: sporadic pancreatic cancer
TJUH: Thomas Jefferson University Hospital

## Notes

^*^ Brown et al., “Identifying a Sequence Alteration in the Regulatory Domain of Cell Cycle Inhibitor, Wee1, Could Define a Novel Familial, Lynch-Like Syndrome and Have Implications for the Treatment of Pancreatic Cancer,” (unpublished data).
